# The Bondons: The Quantum Particles of the Chemical Bond

**DOI:** 10.3390/ijms11114227

**Published:** 2010-10-28

**Authors:** Mihai V. Putz

**Affiliations:** 1 Laboratory of Computational and Structural Physical Chemistry, Chemistry Department, West University of Timişoara, Pestalozzi Street No.16, Timişoara, RO-300115, Romania; E-Mail: mvputz@cbg.uvt.ro or mv_putz@yahoo.com; Tel.: ++40-256-592-633; Fax: ++40-256-592-620; Web: www.mvputz.iqstorm.ro; 2 Theoretical Physics Institute, Free University Berlin, Arnimallee 14, 14195 Berlin, Germany

**Keywords:** de Broglie-Bohm theory, Schrödinger equation, Dirac equation, chemical field, gauge/phase symmetry transformation, bondonic properties, Raman scattering

## Abstract

By employing the combined Bohmian quantum formalism with the U(1) and SU(2) gauge transformations of the non-relativistic wave-function and the relativistic spinor, within the Schrödinger and Dirac quantum pictures of electron motions, the existence of the chemical field is revealed along the associate bondon particle *B̶* characterized by its mass (*m_B̶_*), velocity (*v_B̶_*), charge (*e_B̶_*), and life-time (*t_B̶_*). This is quantized either in ground or excited states of the chemical bond in terms of reduced Planck constant *ħ*, the bond energy *E_bond_* and length *X_bond_*, respectively. The mass-velocity-charge-time quaternion properties of bondons’ particles were used in discussing various paradigmatic types of chemical bond towards assessing their covalent, multiple bonding, metallic and ionic features. The bondonic picture was completed by discussing the relativistic charge and life-time (the actual *zitterbewegung*) problem, *i.e.*, showing that the bondon equals the benchmark electronic charge through moving with almost light velocity. It carries negligible, although non-zero, mass in special bonding conditions and towards observable femtosecond life-time as the bonding length increases in the nanosystems and bonding energy decreases according with the bonding length-energy relationship $E_{\text{bond}}[\text{kcal}/\text{mol}]\times X_{\text{bond}}[\overset{0}{A}]=182019$, providing this way the predictive framework in which the *B̶* particle may be observed. Finally, its role in establishing the virtual states in Raman scattering was also established.

## 1. Introduction

One of the first attempts to systematically use the electron structure as the basis of the chemical bond is due to the discoverer of the electron itself, J.J. Thomson, who published in 1921 an interesting model for describing one of the most puzzling molecules of chemistry, the benzene, by the aid of C–C portioned bonds, each with three electrons [1] that were further separated into 2(σ) + 1(π) lower and higher energy electrons, respectively, in the light of Hückel σ-π and of subsequent quantum theories [2,3]. On the other side, the electronic theory of the valence developed by Lewis in 1916 [4] and expanded by Langmuir in 1919 [5] had mainly treated the electronic behavior like a point-particle that nevertheless embodies considerable chemical information, due to the the semiclassical behavior of the electrons on the valence shells of atoms and molecules. Nevertheless, the consistent quantum theory of the chemical bond was advocated and implemented by the works of Pauling [6–8] and Heitler and London [9], which gave rise to the wave-function characterization of bonding through the fashioned molecular wave-functions (orbitals)–mainly coming from the superposition principle applied on the atomic wave-functions involved. The success of this approach, especially reported by spectroscopic studies, encouraged further generalization toward treating more and more complex chemical systems by the self-consistent wave-function algorithms developed by Slater [10,11], Hartree-Fock [12], Lowdin [13–15], Roothann [16], Pariser, Parr and Pople (in PPP theory) [17–19], until the turn towards the density functional theory of Kohn [20,21] and Pople [22,23] in the second half of the XX century, which marked the subtle feed-back to the earlier electronic point-like view by means of the electronic density functionals and localization functions [24,25]. The compromised picture of the chemical bond may be widely comprised by the emerging Bader’s atoms-in-molecule theory [26–28], the fuzzy theory of Mezey [29–31], along with the chemical reactivity principles [32–43] as originating in the Sanderson’s electronegativity [34] and Pearson’s chemical hardness [38] concepts, and their recent density functionals [44–46] that eventually characterizes it.

Within this modern quantum chemistry picture, its seems that the Dirac dream [47] in characterizing the chemical bond (in particular) and the chemistry (in general) by means of the chemical field related with the Schrödinger wave-function [48] or the Dirac spinor [49] was somehow avoided by collapsing the undulatory quantum concepts into the (observable) electronic density. Here is the paradoxical point: the dispersion of the wave function was replaced by the delocalization of density and the chemical bonding information is still beyond a decisive quantum clarification. Moreover, the quantum theory itself was challenged as to its reliability by the Einstein-Podolski-Rosen(-Bohr) entanglement formulation of quantum phenomena [50,51], qualitatively explained by the Bohm reformulation [52,53] of the de Broglie wave packet [54,55] through the combined de Broglie-Bohm wave-function [56,57]

(1) $$\Psi_{0}(t,x)=R(t,x)\exp\left(i\frac{S(t,x)}{\hbar }\right)$$

with the *R*-amplitude and *S*-phase action factors given, respectively, as

(2) $$R(t,x)=\text{​}\sqrt{\Psi_{0}{\left(t,x\right)}^{2}}=\rho^{1/2}(x)$$

(3) S(t,x)=px−Et

in terms of electronic density ρ, momentum *p*, total energy *E*, and time-space (*t*, *x*) coordinates, without spin.

On the other side, although many of the relativistic effects were explored by considering them in the self-consistent equation of atomic and molecular structure computation [58–62], the recent reloaded thesis of Einstein’s special relativity [63,64] into the algebraic formulation of chemistry [65–67], widely asks for a further reformation of the chemical bonding quantum-relativistic vision [68].

In this respect, the present work advocates making these required steps toward assessing the quantum particle of the chemical bond as based on the derived chemical field released at its turn by the fundamental electronic equations of motion either within Bohmian non-relativistic (Schrödinger) or relativistic (Dirac) pictures and to explore the first consequences. If successful, the present endeavor will contribute to celebrate the dream in unifying the quantum and relativistic features of electron at the chemical level, while unveiling the true particle-wave nature of the chemical bond.

## 2. Method: Identification of Bondons (*B̶*)

The search for the bondons follows the algorithm:

- Considering the de Broglie-Bohm electronic wave-function/spinor Ψ_0_ formulation of the associated quantum Schrödinger/Dirac equation of motion.
- Checking for recovering the charge current conservation law
  (4) $$\frac{\partial \rho }{\partial t}+\nabla \vec{j}=0$$
  that assures for the circulation nature of the electronic fields under study.
- Recognizing the quantum potential *V_qua_* and its equation, if it eventually appears.
- Reloading the electronic wave-function/spinor under the augmented U(1) or SU(2) group form
  (5) $$\Psi_{G}(t,x)=\Psi_{0}(t,x)\exp\left(\frac{i}{\hbar }\frac{e}{c}ℵ(t,x)\right)$$
  with the standard abbreviation $e=e_{0}^{2}/4\pi {ɛ}_{0}$ in terms of the chemical field ℵ considered as the inverse of the fine-structure order:
  (6) $${ℵ}_{0}=\frac{\hbar c}{e}∼137.03599976\left[\frac{\text{Joule}\times \text{meter}}{\text{Coulomb}}\right]$$
  since upper bounded, in principle, by the atomic number of the ultimate chemical stable element (Z = 137). Although apparently small enough to be neglected in the quantum range, the quantity (6) plays a crucial role for chemical bonding where the energies involved are around the order of 10^–19^ Joules (electron-volts)! Nevertheless, for establishing the physical significance of such chemical bonding quanta, one can proceed with the chain equivalences
  (7) $${ℵ}_{\text{B̶}}∼\frac{\text{energy}\times \text{distance}}{\text{charge}}∼\frac{\left(\begin{array}{l} \text{charge}\times \text{potential difference} \end{array}\right)\times \text{distance}}{\text{charge}}∼\left(\begin{array}{l} \text{potential}\\ \text{difference} \end{array}\right)\times \text{distance}$$
  revealing that the chemical bonding field caries *bondons* with unit quanta *ħc*/*e* along the distance of bonding within the potential gap of stability or by tunneling the potential barrier of encountered bonding attractors.
- Rewriting the quantum wave-function/spinor equation with the group object Ψ_G_, while separating the terms containing the real and imaginary ℵ chemical field contributions.
- Identifying the chemical field charge current and term within the actual group transformation context.
- Establishing the global/local gauge transformations that resemble the de Broglie-Bohm wave-function/spinor ansatz Ψ_0_ of steps (i)–(iii).
- Imposing invariant conditions for Ψ*_G_* wave function on pattern quantum equation respecting the Ψ_0_ wave-function/spinor action of steps (i)–(iii).
- Establishing the chemical field ℵ specific equations.
- Solving the system of chemical field ℵ equations.
- Assessing the stationary chemical field
  (8) $$\frac{\partial ℵ}{\partial t}\equiv \partial_{t}ℵ=0$$
  that is the case in chemical bonds at equilibrium (ground state condition) to simplify the quest for the solution of chemical field ℵ.
- The manifested bondonic chemical field ℵ*_bondon_* is eventually identified along the bonding distance (or space).
- Checking the eventual charge flux condition of Bader within the vanishing chemical bonding field [26]
  (9) $${ℵ}_{\text{B̶}}=0\Leftrightarrow \nabla \rho =0$$
- Employing the Heisenberg time-energy relaxation-saturation relationship through the kinetic energy of electrons in bonding
  (10) $$v=\sqrt{\frac{2T}{m}}∼\sqrt{\frac{2}{m}\frac{\hbar }{t}}$$
- Equate the bondonic chemical bond field with the chemical field quanta (6) to get the bondons’ mass
  (11) $${ℵ}_{\text{B̶}}(m_{\text{B̶}})={ℵ}_{0}$$

This algorithm will be next unfolded both for non-relativistic as well as for relativistic electronic motion to quest upon the bondonic existence, eventually emphasizing their difference in bondons’ manifestations.

## 3. Type of Bondons

### 3.1. Non-Relativistic Bondons

For the non-relativistic quantum motion, we will treat the above steps (i)–(iii) at once. As such, when considering the de Broglie-Bohm electronic wavefunction into the Schrödinger Equation [48]

(12) $$i\hbar \partial_{t}\Psi_{0}=-\frac{\hbar^{2}}{2m}\nabla^{2}\Psi_{0}+V\Psi_{0}$$

it separates into the real and imaginary components as [52,53,68]

(13a) $$\partial_{t}R^{2}+\nabla \left(\frac{R^{2}}{m}\nabla S\right)=0$$

(13b) $$\partial_{t}S-\frac{\hbar^{2}}{2m}\frac{1}{R}\nabla^{2}R+\frac{1}{2m}{\left(\nabla S\right)}^{2}+V=0$$

While recognizing into the first Equation (13a), the charge current conservation law with Equation (2) along the identification

(14) $${\vec{j}}_{S}=\frac{R^{2}}{m}\nabla S$$

the second equation helps in detecting the quantum (or Bohm) potential

(15) $$V_{\text{qua}}=-\frac{\hbar^{2}}{2m}\frac{\nabla^{2}R}{R}$$

contributing to the total energy

(16) $$E=T+V+V_{\text{qua}}$$

once the momentum-energy correspondences

(17a) $$\frac{1}{2m}{\left(\nabla S\right)}^{2}=\frac{p^{2}}{2m}=T$$

(17b) $$\partial_{t}S=-E$$

are engaged.

Next, when employing the associate U(1) gauge wavefunction of Equation (5) type, its partial derivative terms look like

(18a) $$\nabla \Psi_{G}=\left[\nabla R+\frac{i}{\hbar }R\left(\nabla S+\frac{e}{c}\nabla ℵ\right)\right]\exp\left[\frac{i}{\hbar }\left(S+\frac{e}{c}ℵ\right)\right]$$

(18b) $$\nabla^{2}\Psi_{G}=\left\{\begin{array}{l} \nabla^{2}R+2\frac{i}{\hbar }\nabla R\left(\nabla S+\frac{e}{c}\nabla ℵ\right)+\frac{i}{\hbar }R\left(\nabla^{2}S+\frac{e}{c}\nabla^{2}ℵ\right)\\ -\frac{R}{\hbar^{2}}\left[{\left(\nabla S\right)}^{2}+{\left(\frac{e}{c}\nabla ℵ\right)}^{2}\right]-2\frac{e}{\hbar^{2}c}R\nabla S\nabla ℵ \end{array}\right\}\exp\left[\frac{i}{\hbar }\left(S+\frac{e}{c}ℵ\right)\right]$$

(18c) $$\partial_{t}\Psi_{G}=\left[\partial_{t}R+\frac{i}{\hbar }R\left(\partial_{t}S+\frac{e}{c}\partial_{t}ℵ\right)\right]\exp\left[\frac{i}{\hbar }\left(S+\frac{e}{c}ℵ\right)\right]$$

Now the Schrödinger Equation (12) for Ψ*_G_* in the form of (5) is decomposed into imaginary and real parts

(19a) $$-\partial_{t}R=\frac{1}{m}\left(\nabla R\cdot \nabla S+\frac{R}{2}\nabla^{2}S\right)+\frac{e}{\text{mc}}\left(\nabla R\cdot \nabla ℵ+\frac{R}{2}\nabla^{2}ℵ\right)$$

(19b) $$-R\partial_{t}S-R\frac{e}{c}\partial_{t}ℵ=-\frac{\hbar^{2}}{2m}\nabla^{2}R+\frac{R}{2m}\left[{\left(\nabla S\right)}^{2}+{\left(\frac{e}{c}\nabla ℵ\right)}^{2}\right]+\frac{e}{\text{mc}}R\nabla S\cdot \nabla ℵ+VR$$

that can be rearranged

(20a) $$-\partial_{t}R^{2}=\frac{1}{m}\nabla \left(R^{2}\nabla S\right)+\frac{e}{\text{mc}}\nabla \left(R^{2}\nabla ℵ\right)$$

(20b) $$-\left(\partial_{t}S+\frac{e}{c}\partial_{t}ℵ\right)=-\frac{\hbar^{2}}{2m}\frac{1}{R}\nabla^{2}R+\frac{1}{2m}\left[{\left(\nabla S\right)}^{2}+{\left(\frac{e}{c}\nabla ℵ\right)}^{2}\right]+\frac{e}{\text{mc}}\nabla S.\nabla ℵ+V$$

to reveal some interesting features of chemical bonding.

Firstly, through comparing the Equation (20a) with the charge conserved current equation form (4) from the general chemical field algorithm–the step (ii), the conserving charge current takes now the expanded expression:

(21) $${\vec{j}}_{U(1)}=\frac{R^{2}}{m}\left(\nabla S+\frac{e}{c}\nabla ℵ\right)={\vec{j}}_{S}+{\vec{j}}_{ℵ}$$

suggesting that the additional current is responsible for the chemical field to be activated, namely

(22) $${\vec{j}}_{ℵ}=\frac{e}{\text{mc}}R^{2}\nabla ℵ$$

which vanishes when the *global gauge* condition is considered

(23) ∇ℵ=0

Therefore, in order that the chemical bonding is created, the *local gauge* transformation should be used that exists under the condition

(24) ∇ℵ≠0

In this framework, the chemical field current *j⃗*_ℵ_ carries specific bonding particles that can be appropriately called *bondons*, closely related with electrons, in fact with those electrons involved in bonding, either as single, lone pair or delocalized, and having an oriented direction of movement, with an action depending on the chemical field itself ℵ.

Nevertheless, another important idea abstracted from the above results is that in the search for the chemical field ℵ no global gauge condition is required. It is also worth noting that the presence of the chemical field does not change the Bohm quantum potential that is recovered untouched in (20b), thus preserving the entanglement character of interaction.

With these observations, it follows that in order for the de Broglie-Bohm-Schrödinger formalism to be invariant under the U(1) transformation (5), a couple of gauge conditions have to be fulfilled by the chemical field in Equations (20a) and (20b), namely

(25a) $$\frac{e}{\text{mc}}\frac{\partial }{\partial x}\left(R^{2}\nabla ℵ\right)=0$$

(25b) $$\frac{e}{c}\partial_{t}ℵ+\frac{1}{2m}{\left(\frac{e}{c}\nabla ℵ\right)}^{2}+\frac{e}{\text{mc}}\nabla S.\nabla ℵ=0$$

Next, the chemical field ℵ is to be expressed through combining its spatial-temporal information contained in Equations (25). From the first condition (25a) one finds that

(26) $$\nabla ℵ=\frac{R}{2}\frac{\nabla^{2}ℵ}{\nabla R\cdot \vec{ı}}\vec{ı}$$

where the vectorial feature of the chemical field gradient was emphasized on the direction of its associated charge current fixed by the versor *i⃗* (*i.e.*, by the unitary vector associate with the propagation direction, *i⃗*^2^=1). We will apply such writing whenever necessary for avoiding scalar to vector ratios and preserving the physical sense of the whole construction as well. Replacing the gradient of the chemical field (26) into its temporal Equation (25b) one gets the unified chemical field motion description

(27) $$\frac{e}{8\text{mc}}\frac{R^{2}}{{\left(\nabla R\right)}^{2}}{\left(\nabla^{2}ℵ\right)}^{2}-\frac{R}{2m}\frac{\nabla S\cdot \nabla S}{\nabla R\cdot \nabla S}\left(\nabla^{2}ℵ\right)+\partial_{t}ℵ=0$$

that can be further rewritten as

(28) $$\frac{e}{2\text{mc}}\frac{\rho }{{\left(\nabla \rho \right)}^{2}}{\left(\nabla^{2}ℵ\right)}^{2}-\frac{\rho \vec{\nu }\cdot \vec{ı}}{\nabla \rho \cdot \vec{ı}}\left(\nabla^{2}ℵ\right)+\partial_{t}ℵ=0$$

since calling the relations abstracted from Equations (2) and (3)

(29) $$R=\rho^{1/2};\nabla S=\vec{p}\Rightarrow \{\begin{array}{l} \nabla R=\frac{1}{2}\frac{\nabla \rho }{\rho^{1/2}};{\left(\nabla R\right)}^{2}=\frac{1}{4}\frac{{\left(\nabla \rho \right)}^{2}}{\rho }\\ \frac{\nabla S\cdot \nabla S}{\nabla R\cdot \nabla S}=\frac{2\rho^{1/2}\vec{p}\cdot \vec{ı}}{\nabla \rho \cdot \vec{ı}} \end{array}$$

The (quadratic undulatory) chemical field Equation (28) can be firstly solved for the Laplacian general solutions

(30) $${\left(\nabla^{2}ℵ\right)}_{1,2}=\frac{\frac{\rho \vec{\nu }\cdot \vec{ı}}{\nabla \rho \cdot \vec{ı}}\pm \sqrt{\frac{\rho^{2}\nu^{2}}{{\left(\nabla \rho \right)}^{2}}-\frac{2e}{\text{mc}}\frac{\rho^{2}}{{\left(\nabla \rho \right)}^{2}}\partial_{t}ℵ}}{\frac{e}{\text{mc}}\frac{\rho^{2}}{{\left(\nabla \rho \right)}^{2}}}$$

that give special propagation equations for the chemical field since linking the spatial Laplacian with temporal evolution of the chemical field (∂*_t_*ℵ)^1/2^; however, they may be considerably simplified when assuming the stationary chemical field condition (8), the step (xi) in the bondons’ algorithm, providing the working equation for the stationary bondonic field

(31) $$\nabla^{2}ℵ=2\frac{\text{mc}}{e}\frac{\vec{\nu }\cdot \nabla \rho }{\rho }$$

Equation (31) may be further integrated between two bonding attractors, say *X_A_*,*X_B_*, to primarily give

(32) $$\nabla ℵ=2\frac{\text{mc}}{e}\nu \int_{X_{A}}^{X_{B}}\frac{\nabla \rho \cdot \vec{ı}}{\rho }dx=\frac{\text{mc}}{e}\nu \left[\int_{X_{A}}^{X_{B}}\frac{\nabla \rho \cdot \vec{ı}}{\rho }dx-\int_{X_{B}}^{X_{A}}\frac{\nabla \rho \cdot \vec{ı}}{\rho }dx\right]$$

from where the generic bondonic chemical field is manifested with the form

(33) $${ℵ}_{\text{B̶}}=\frac{\text{mc}}{e}\nu X_{\text{bond}}\left(\int_{X_{A}}^{X_{B}}\frac{\nabla \rho \cdot \vec{ı}}{\rho }dx\right)$$

The expression (33) has two important consequences. Firstly, it recovers the Bader zero flux condition for defining the *basins* of bonding [26] that in the present case is represented by the zero chemical boning fields, namely

(34) $${ℵ}_{\text{B̶}}=0\Leftrightarrow \nabla \rho \cdot \vec{ı}=0$$

Secondly, it furnishes the bondonic (chemical field) analytical expression

(35) $${ℵ}_{\text{B̶}}=\frac{\text{mc}}{e}\nu X_{\text{bond}}$$

within the natural framework in which

(36a) $$X_{B}-X_{A}=X_{\text{bond}}$$

(36b) $$\nabla \rho \cdot \vec{ı}\to \frac{\rho }{X_{\text{bond}}}$$

*i.e.*, when one has

(36c) $$\int_{X_{A}}^{X_{B}}\frac{\nabla \rho \cdot \vec{ı}}{\rho }dx=1$$

The step (xiv) of the bondonic algorithm may be now immediately implemented through inserting the Equation (10) into Equation (35) yielding the simple chemical field form

(37) $${ℵ}_{\text{B̶}}=\frac{c\hbar }{e}\sqrt{\frac{2m}{\hbar t}}X_{\text{bond}}$$

Finally, through applying the expression (11) of the bondonic algorithm–the step (xv) upon the result (37) with quanta (6) the *mass of bondons* carried by the chemical field on a given distance is obtained

(38) $$m_{\text{B̶}}=\frac{\hbar t}{2}\frac{1}{X_{\text{bond}}^{2}}$$

Note that the bondons’ mass (38) directly depends on the time the chemical information “travels” from one bonding attractor to the other involved in bonding, while fast decreasing as the bonding distance increases. This phenomenological behavior has to be in the sequel cross-checked by considering the generalized relativistic version of electronic motion by means of the Dirac equation, Further quantitative consideration will be discussed afterwards.

### 3.2. Relativistic Bondons

In treating the quantum relativistic electronic behavior, the consecrated starting point stays the Dirac equation for the scalar real valued potential *w* that can be seen as a general function of (*tc*,*x⃗*) dependency [49]

(39) $$i\hbar \partial_{t}{\overset{↔}{\Psi }}_{0}=\left[-i\hbar c\sum_{k=1}^{3}{\hat{\alpha }}_{k}\partial_{k}+\hat{\beta }mc^{2}+\hat{\beta }w\right]{\overset{↔}{\Psi }}_{0}$$

with the spatial coordinate derivative notation ∂*_k_* ≡ ∂/∂*x_k_* and the special operators assuming the Dirac 4D representation

(40a) $${\hat{\alpha }}_{k}=\left[\begin{array}{cc} 0 & {\hat{\sigma }}_{k}\\ {\hat{\sigma }}_{k} & 0 \end{array}\right],\hat{\beta }=\left[\begin{array}{cc} \hat{1} & 0\\ 0 & -\hat{1} \end{array}\right]$$

in terms of bi-dimensional Pauli and unitary matrices

(40b) $${\hat{\sigma }}_{1}=\left[\begin{array}{cc} 0 & 1\\ 1 & 0 \end{array}\right],\;\;{\hat{\sigma }}_{2}=\left[\begin{array}{cc} 0 & -i\\ i & 0 \end{array}\right],\;\;{\hat{\sigma }}_{3}=\left[\begin{array}{cc} 1 & 0\\ 0 & -1 \end{array}\right],\;\;\hat{1}\equiv {\hat{\sigma }}_{0}=\left[\begin{array}{cc} 1 & 0\\ 0 & 1 \end{array}\right]$$

Written within the de Broglie-Bohm framework, the spinor solution of Equation (39) looks like

(41) $${\overset{↔}{\Psi }}_{0}=\frac{1}{\sqrt{2}}R(t,x)\left[\begin{array}{c} \phi \\ \varphi \end{array}\right]=\frac{1}{\sqrt{2}}R(t,x)\left[\begin{array}{c} \text{exp}\left\{\frac{i}{\hbar }\left[S\left(t,x\right)+s\right]\right\}\\ \text{exp}\left\{-\frac{i}{\hbar }\left[S\left(t,x\right)+s\right]\right\} \end{array}\right],\;\;s=\pm \frac{1}{2}$$

that from the beginning satisfies the necessary electronic density condition

(42) $${\overset{↔}{\Psi }}_{0}^{*}{\overset{↔}{\Psi }}_{0}=R^{*}R=\rho$$

Going on, aiming for the separation of the Dirac Equation (39) into its real/imaginary spinorial contributions, one firstly calculates the terms

(43a) $$\partial_{t}{\overset{↔}{\Psi }}_{0}=\frac{1}{\sqrt{2}}\partial_{t}R\left[\begin{array}{c} \varphi \\ \phi \end{array}\right]+\frac{1}{\sqrt{2}}R\frac{i}{\hbar }\partial_{t}S\left[\begin{array}{c} \varphi \\ -\phi \end{array}\right]$$

(43b) $$\partial_{t}{\overset{↔}{\Psi }}_{0}=\frac{1}{\sqrt{2}}\partial_{k}R\left[\begin{array}{c} \varphi \\ \phi \end{array}\right]+\frac{1}{\sqrt{2}}R\frac{i}{\hbar }\partial_{k}S\left[\begin{array}{c} \varphi \\ -\phi \end{array}\right]$$

(43c) $$\begin{array}{ll} \sum_{k=1}^{3}{\hat{\alpha }}_{k}\partial_{k}{\overset{↔}{\Psi }}_{0} & =\frac{1}{\sqrt{2}}\sum_{k=1}^{3}\partial_{k}R\left[\begin{array}{cc} 0 & {\hat{\sigma }}_{k}\\ {\hat{\sigma }}_{k} & 0 \end{array}\right]\left[\begin{array}{c} \varphi \\ \phi \end{array}\right]+\frac{1}{\sqrt{2}}R\frac{i}{\hbar }\sum_{k=1}^{3}\partial_{k}S\left[\begin{array}{cc} 0 & {\hat{\sigma }}_{k}\\ {\hat{\sigma }}_{k} & 0 \end{array}\right]\left[\begin{array}{c} \varphi \\ -\phi \end{array}\right]\\ & =\frac{1}{\sqrt{2}}\left[\begin{array}{c} \phi \sum_{k}\left(\partial_{k}R\right){\hat{\sigma }}_{k}\\ \varphi \sum_{k}\left(\partial_{k}R\right){\hat{\sigma }}_{k} \end{array}\right]+\frac{1}{\sqrt{2}}R\frac{i}{\hbar }\left[\begin{array}{c} -\phi \sum_{k}\left(\partial_{k}S\right){\hat{\sigma }}_{k}\\ \varphi \sum_{k}\left(\partial_{k}S\right){\hat{\sigma }}_{k} \end{array}\right] \end{array}$$

(43d) $$\hat{\beta }mc^{2}{\overset{↔}{\Psi }}_{0}=\frac{mc^{2}}{\sqrt{2}}R\left[\begin{array}{cc} \hat{1} & 0\\ 0 & -\hat{1} \end{array}\right]\left[\begin{array}{c} \varphi \\ \phi \end{array}\right]=\frac{mc^{2}}{\sqrt{2}}R\left[\begin{array}{c} \varphi \\ -\phi \end{array}\right]$$

(43e) $$\hat{\beta }w{\overset{↔}{\Psi }}_{0}=\frac{w}{\sqrt{2}}R\left[\begin{array}{c} \varphi \\ -\phi \end{array}\right]$$

to be then combined in (39) producing the actual de Broglie-Bohm-Dirac spinorial Equation

(44) $$\left[\begin{array}{c} i\hbar \varphi \partial_{t}R-R\varphi \partial_{t}S\\ i\hbar \phi \partial_{t}R+R\phi \partial_{t}S \end{array}\right]=\left[\begin{array}{c} -i\hbar c\phi \sum_{k}\left(\partial_{k}R\right){\hat{\sigma }}_{k}-Rc\phi \sum_{k}\left(\partial_{k}S\right){\hat{\sigma }}_{k}+\left(mc^{2}+w\right)R\varphi \\ -i\hbar c\varphi \sum_{k}\left(\partial_{k}R\right){\hat{\sigma }}_{k}+Rc\varphi \sum_{k}\left(\partial_{k}S\right){\hat{\sigma }}_{k}-\left(mc^{2}+w\right)R\phi \end{array}\right]$$

When equating the imaginary parts of (44) one yields the system

(45) $$\{\begin{array}{l} \varphi \partial_{t}R+\phi c\sum_{k}\left(\partial_{k}R\right){\hat{\sigma }}_{k}=0\\ \varphi c\sum_{k}\left(\partial_{k}R\right){\hat{\sigma }}_{k}+\phi \partial_{t}R=0 \end{array}$$

that has non-trivial spinorial solutions only by canceling the associate determinant, *i.e.*, by forming the Equation

(46) $${\left(\partial_{t}R\right)}^{2}=c^{2}{\left[\sum_{k}\left(\partial_{k}R\right){\hat{\sigma }}_{k}\right]}^{2}$$

of which the minus sign of the squared root corresponds with the electronic conservation charge, while the positive sign is specific to the relativistic treatment of the positron motion. For proofing this, the specific relationship for the electronic charge conservation (4) may be unfolded by adapting it to the present Bohmian spinorial case by the chain equivalences

(47a) $$\begin{array}{c} 0=\partial_{t}\rho +\nabla \vec{j}\\ =\partial_{t}\left(R^{2}\right)+\sum_{k}\partial_{k}j_{k}\\ =2R\partial_{t}R+\sum_{k}\partial_{k}\left(c{\overset{↔}{\Psi }}_{0}^{*}{\hat{\alpha }}_{k}{\overset{↔}{\Psi }}_{0}\right)\\ =2R\partial_{t}R+\frac{c}{2}\sum_{k}\partial_{k}R^{*}R\left[e^{-\frac{i}{\hbar }(S+s)}\;e^{\frac{i}{\hbar }(S+s)}\right]\left[\begin{array}{cc} 0 & {\hat{\sigma }}_{k}\\ {\hat{\sigma }}_{k} & 0 \end{array}\right]\left[\begin{array}{c} e^{\frac{i}{\hbar }(S+s)}\\ e^{-\frac{i}{\hbar }(S+s)} \end{array}\right]\\ =2R\partial_{t}R+\frac{c}{2}\sum_{k}{\hat{\sigma }}_{k}(\underset{1}{\underset{︸}{\varphi^{2}}}+\underset{1}{\underset{︸}{\phi^{2}}})\partial_{k}R^{2}\\ =2R\partial_{t}R+2\text{Rc}\sum_{k}{\hat{\sigma }}_{k}\left(\partial_{k}R\right) \end{array}$$

The result

(47b) $$\partial_{t}R=-c\sum_{k}{\hat{\sigma }}_{k}\left(\partial_{k}R\right)$$

indeed corresponds with the squaring root of (46) with the minus sign, certifying, therefore, the validity of the present approach, *i.e.*, being in accordance with the step (ii) in bondonic algorithm of Section 2.

Next, let us see what information is conveyed by the real part of Bohmian decomposed spinors of Dirac Equation (44); the system (48) is obtained

(48) $$\{\begin{array}{l} \varphi \left(\partial_{t}S+mc^{2}+w\right)-\phi c\sum_{k}\left(\partial_{k}S\right){\hat{\sigma }}_{k}=0\\ \varphi c\sum_{k}\left(\partial_{k}S\right){\hat{\sigma }}_{k}-\left(\partial_{t}S+mc^{2}+w\right)\phi =0 \end{array}$$

that, as was previously the case with the imaginary counterpart (45), has no trivial spinors solutions only if the associate determinant vanishes, which gives the Equation

(49) $$c^{2}{\left[\sum_{k}\left(\partial_{k}S\right){\hat{\sigma }}_{k}\right]}^{2}={\left(\partial_{t}S+mc^{2}+w\right)}^{2}$$

Now, considering the Bohmian momentum-energy (17) equivalences, the Equation (49) further becomes

(50) $$\begin{array}{l} c^{2}{\left[\sum_{k}p_{k}{\hat{\sigma }}_{k}\right]}^{2}={\left(-E+mc^{2}+w\right)}^{2}\\ \Leftrightarrow c^{2}{\left(\vec{p}\cdot \hat{\vec{\sigma }}\right)}^{2}={\left(-E+mc^{2}+w\right)}^{2}\\ \Leftrightarrow c^{2}p^{2}={\left(-E+mc^{2}+w\right)}^{2} \end{array}$$

from where, while retaining the minus sign through the square rooting (as prescribed above by the imaginary spinorial treatment in relation with charge conservation), one recovers the relativistic electronic energy-momentum conservation relationship

(51) $$E=cp+mc^{2}+w$$

thus confirming in full the reliability of the Bohmian approach over the relativistic spinors.

Moreover, the present Bohmian treatment of the relativistic motion is remarkable in that, except in the non-relativistic case, it does not produces the additional quantum (Bohm) potential (15)–responsible for entangled phenomena or hidden variables. This may be justified because within the Dirac treatment of the electron the entanglement phenomenology is somehow included throughout the Dirac Sea and the positron existence. Another important difference with respect to the Schrödinger picture is that the spinor equations that underlie the total charge and energy conservation do not mix the amplitude (2) with the phase (3) of the de Broglie-Bohm wave-function, whereas they govern now, in an independent manner, the flux and the energy of electronic motion. For these reasons, it seems that the relativistic Bohmian picture offers the natural environment in which the chemical field and associate bondons particles may be treated without involving additional physics.

Let us see, therefore, whether the Dirac-Bohmian framework will reveal (or not) new insight in the bondon (Schrödinger) reality. This will be done by reconsidering the working Bohmian spinor (41) as transformed by the internal gauge symmetry SU(2) driven by the chemical field ℵ related phase–in accordance with Equation (5) of the step (iv) of bondonic algorithm

(52) $$\begin{array}{c} {\overset{↔}{\Psi }}_{G}(t,x)={\overset{↔}{\Psi }}_{0}(t,x)\text{exp}\left(\frac{i}{\hbar }\frac{e}{c}ℵ(t,x)\right)\\ =\frac{1}{\sqrt{2}}R(t,x)\left[\begin{array}{l} \varphi_{G}\\ \phi_{G} \end{array}\right]=\frac{1}{\sqrt{2}}R(t,x)\left[\begin{array}{l} \text{exp}\left\{\frac{i}{\hbar }\left[s(t,x)+\frac{e}{c}ℵ(t,x)+s\right]\right\}\\ \text{exp}\left\{-\frac{i}{\hbar }\left[s(t,x)+\frac{e}{c}ℵ(t,x)+s\right]\right\} \end{array}\right] \end{array}$$

Here it is immediate that expression (52) still preserves the electronic density formulation (2) as was previously the case with the gaugeless field (41)

(53) $${\overset{↔}{\Psi }}_{G}^{*}{\overset{↔}{\Psi }}_{G}=R*R=\rho$$

However, when employed for the Dirac equation terms, the field (52) modifies the previous expressions (43a)–(43c) as follows

(54a) $$\partial_{t}{\overset{↔}{\Psi }}_{G}=\frac{1}{\sqrt{2}}\partial_{t}R\left[\begin{array}{l} \varphi_{G}\\ \phi_{G} \end{array}\right]+\frac{1}{\sqrt{2}}R\frac{i}{\hbar }\left(\partial_{t}S+\frac{e}{c}\partial_{t}ℵ\right)\left[\begin{array}{l} \varphi_{G}\\ -\phi_{G} \end{array}\right]$$

(54b) $$\partial_{k}{\overset{↔}{\Psi }}_{0}=\frac{1}{\sqrt{2}}\partial_{k}R\left[\begin{array}{l} \varphi_{G}\\ \phi_{G} \end{array}\right]+\frac{1}{\sqrt{2}}R\frac{i}{\hbar }\left(\partial_{k}S+\frac{e}{c}\partial_{k}ℵ\right)\left[\begin{array}{l} \varphi_{G}\\ -\phi_{G} \end{array}\right]$$

(54c) $$\sum_{k=1}^{3}{\hat{\alpha }}_{k}\partial_{k}{\overset{↔}{\Psi }}_{G}=\frac{1}{\sqrt{2}}\sum_{k}(\partial_{k}R){\hat{\sigma }}_{k}\left[\begin{array}{l} \phi_{G}\\ \varphi_{G} \end{array}\right]+\frac{1}{\sqrt{2}}R\frac{i}{\hbar }\sum_{k}\left(\partial_{k}S+\frac{e}{c}\partial_{k}ℵ\right){\hat{\sigma }}_{k}\left[\begin{array}{l} -\phi_{G}\\ \varphi_{G} \end{array}\right]$$

while producing the gauge spinorial Equation

(55) [iħφG∂tR−RφG(∂tS+ec∂tℵ)iħϕG∂tR+RϕG(∂tS+ec∂tℵ)]=[−iħcϕG∑k(∂kR)σ^k−RcϕG∑k(∂kS+ec∂kℵ)σ^k+(mc2+w)RφG−iħcφG∑k(∂kR)σ^k−RcφG∑k(∂kS+ec∂kℵ)σ^k+(mc2+w)RϕG]

Now it is clear that since the imaginary part in (55) was not at all changed with respect to Equation (44) by the chemical field presence, the total charge conservation (4) is naturally preserved; instead the real part is modified, respecting the case (44), in the presence of the chemical field (by internal gauge symmetry). Nevertheless, in order that chemical field rotation does not produce modification in the total energy conservation, it imposes that the gauge spinorial system of the chemical field must be as

(56) $$\left\{ \begin{array}{l} \varphi_{G}\partial_{t}ℵ-\phi_{G}c\sum_{k}(\partial_{k}ℵ){\hat{\sigma }}_{k}=0\\ \varphi_{G}c\sum_{k}(\partial_{k}ℵ){\hat{\sigma }}_{k}-\phi_{G}\partial_{t}ℵ=0 \end{array}\right.$$

According to the already custom procedure, for the system (56) having no trivial gauge spinorial solution, the associated vanishing determinant is necessary, which brings to light the chemical field Equation

(57a) $$c^{2}{\left[\sum_{k}\left(\partial_{k}ℵ\right){\hat{\sigma }}_{k}\right]}^{2}={\left(\partial_{t}ℵ\right)}^{2}$$

equivalently rewritten as

(57b) $$c^{2}{\left[\nabla ℵ\cdot \hat{\vec{\sigma }}\right]}^{2}={\left(\partial_{t}ℵ\right)}^{2}$$

that simply reduces to

(57c) $$c^{2}{\left(\nabla ℵ\right)}^{2}={\left(\partial_{t}ℵ\right)}^{2}$$

through considering the Pauling matrices (40b) unitary feature upon squaring.

At this point, one has to decide upon the sign of the square root of (57c); this was previously clarified to be minus for electronic and plus for positronic motions. Therefore, the electronic chemical bond is modeled by the resulting chemical field equation projected on the bonding length direction

(58) $$\frac{\partial ℵ}{\partial X_{\text{bond}}}=\frac{1}{c}\frac{\partial ℵ}{\partial t}$$

The Equation (58) is of undulatory kind with the chemical field solution having the general plane wave form

(59) $${ℵ}_{\text{B̶}}=\frac{\hbar c}{e}\text{exp}\left[i(kX_{\text{bond}}-\omega t)\right]$$

that agrees with both the general definition of the chemical field (6) as well as with the relativistic “traveling” of the bonding information. In fact, this is the paradox of the Dirac approach of the chemical bond: it aims to deal with electrons in bonding while they have to transmit the chemical bonding information—as waves—propagating with the light velocity between the bonding attractors. This is another argument for the need of bondons reality as a specific existence of electrons in chemical bond is compulsory so that such a paradox can be solved.

Note that within the Dirac approach, the Bader flux condition (9) is no more related to the chemical field, being included in the total conservation of charge; this is again natural, since in the relativistic case the chemical field is explicitly propagating with a percentage of light velocity (see the Discussion in Section 4 below) so that it cannot drive the (stationary) electronic frontiers of bonding.

Further on, when rewriting the chemical field of bonding (59) within the de Broglie and Planck consecrated corpuscular-undulatory quantifications

(60) $${ℵ}_{\text{B̶}}\left(t,\;X_{\text{bond}}\right)=\frac{\hbar c}{e}\text{exp}\left[\frac{i}{\hbar }(pX_{\text{bond}}-Et)\right]$$

it may be further combined with the unitary quanta form (6) in the Equation (11) of the step (xv) in the bondonic algorithm to produce the phase condition

(61) $$1=\text{exp}\left[\frac{i}{\hbar }(pX_{\text{bond}}-Et)\right]$$

that implies the quantification

(62) $$pX_{\text{bond}}-Et=2\pi n\hbar ,\;\;n\in \text{N}$$

By the subsequent employment of the Heisenberg time-energy saturated indeterminacy at the level of kinetic energy abstracted from the total energy (to focus on the motion of the bondonic plane waves)

(63a) $$E=\frac{\hbar }{t}$$

(63b) $$p=mv=\sqrt{2mT}\to \sqrt{\frac{2m\hbar }{t}}$$

the bondon Equation (62) becomes

(64) $$X_{\text{bond}}\sqrt{\frac{2m\hbar }{t}}=(2\pi n+1)\hbar$$

that when solved for the bondonic mass yields the expression

(65) $$m_{\text{B̶}}=\frac{\hbar t}{2}\frac{1}{X_{\text{bond}}^{2}}{\left(2\pi n+1\right)}^{2},\;\;n=0,1,2\ldots$$

which appears to correct the previous non-relativistic expression (38) with the full quantification.

However, the Schrödinger bondon mass of Equation (38) is recovered from the Dirac bondonic mass (65) in the ground state, *i.e.*, by setting *n* = 0. Therefore, the Dirac picture assures the complete characterization of the chemical bond through revealing the bondonic existence by the internal chemical field symmetry with the quantification of mass either in ground or in excited states (*n* ≤ 0, *n* ∈ **N**).

Moreover, as always happens when dealing with the Dirac equation, the positronic bondonic mass may be immediately derived as well, for the case of the chemical bonding is considered also in the anti-particle world; it emerges from reloading the square root of the Dirac chemical field Equation (57c) with a plus sign that will be propagated in all the subsequent considerations, e.g., with the positronic incoming plane wave replacing the departed electronic one of (59), until delivering the positronic bondonic mass

(66) $${\tilde{m}}_{\text{B̶}}=\frac{\hbar t}{2}\frac{1}{X_{\text{bond}}^{2}}{\left(2\pi n-1\right)}^{2},\;\;n=0,1,2\ldots$$

It nevertheless differs from the electronic bondonic mass (65) only in the excited spectrum, while both collapse in the non-relativistic bondonic mass (38) for the ground state of the chemical bond.

Remarkably, for both the electronic and positronic cases, the associated bondons in the excited states display heavier mass than those specific to the ground state, a behavior once more confirming that the bondons encompass all the bonding information, *i.e.*, have the excitation energy converted in the mass-added-value in full agreement with the mass-energy relativistic custom Einstein equivalence [64].

## 4. Discussion

Let us analyze the consequences of the bondon’s existence, starting from its mass (38) formulation on the ground state of the chemical bond.

At one extreme, when considering *atomic* parameters in bonding, *i.e.*, when assuming the bonding distance of the Bohr radius size *a*_0_ = 0.52917 · 10^−10^[*m*]*_SI_* the corresponding binding time would be given as *t* → *t*_0_ = *a*_0_/*v*_0_ = 2.41889 · 10^−17^[*s*]*_SI_* while the involved bondonic mass will be half of the electronic one *m*_0_/2, to assure fast bonding information. Of course, this is not a realistic binding situation; for that, let us check the hypothetical case in which the electronic *m*_0_ mass is combined, within the bondonic formulation (38), into the bond distance $X_{\text{bond}}=\sqrt{\hbar t/2m_{0}}$ resulting in it completing the binding phenomenon in the femtosecond time *t_bonding_* ∼ 10^−12^[*s*]*_SI_* for the custom nanometric distance of bonding *X_bonding_* ∼ 10^−9^[*m*]*_SI_*. Still, when both the femtosecond and nanometer time-space scale of bonding is assumed in (38), the bondonic mass is provided in the range of electronic mass *m_B̶_* ∼ 10^−31^[*kg*]*_SI_* although not necessarily with the exact value for electron mass nor having the same value for each bonding case considered. Further insight into the time existence of the bondons will be reloaded for molecular systems below after discussing related specific properties as the bondonic velocity and charge.

For enlightenment on the last perspective, let us rewrite the bondonic mass (65) within the spatial-energetic frame of bonding, *i.e.*, through replacing the time with the associated Heisenberg energy, *t_bonding_* → *ħ/E_bond_*, thus delivering another working expression for the bondonic mass

(67) $$m_{\text{B̶}}=\frac{\hbar^{2}}{2}\frac{{\left(2\pi n+1\right)}^{2}}{E_{\text{bond}}X_{\text{bond}}^{2}},\;\;n=0,1,2\ldots$$

that is more practical than the traditional characterization of bonding types in terms of length and energy of bonding; it may further assume the numerical ground state ratio form

(68) $$\zeta_{m}=\frac{m_{\text{B̶}}}{m_{0}}=\frac{87.8603}{(E_{\text{bond}}[\text{kcal}/\text{mol}]){\left(X_{\text{bond}}[\overset{0}{A}]\right)}^{2}}$$

when the available bonding energy and length are considered (as is the custom for chemical information) in kcal/mol and Angstrom, respectively. Note that having the bondon’s mass in terms of bond energy implies the inclusion of the electronic pairing effect in the bondonic existence, without the constraint that the bonding pair may accumulate in the internuclear region [69].

Moreover, since the bondonic mass general formulation (65) resulted within the relativistic treatment of electron, it is considering also the companion velocity of the bondonic mass that is reached in propagating the bonding information between the bonding attractors. As such, when the Einstein type relationship [70]

(69) $$\frac{mv^{2}}{2}=h\upsilon$$

is employed for the relativistic bondonic velocity-mass relationship [63,64]

(70) $$m=\frac{m_{\text{B̶}}}{\sqrt{1-\frac{v_{\text{B̶}}^{2}}{c^{2}}}}$$

and for the frequency of the associate bond wave

(71) $$\upsilon =\frac{v_{\text{B̶}}}{X_{\text{bond}}}$$

it provides the quantified searched bondon to light velocity ratio

(72) $$\frac{v_{\text{B̶}}}{c}=\frac{1}{\sqrt{1+\frac{1}{64\pi^{2}}\frac{\hbar^{2}c^{2}{\left(2\pi n+1\right)}^{4}}{E_{\text{bond}}^{2}X_{\text{bond}}^{2}}}},\;n=0,1,2\ldots$$

or numerically in the bonding ground state as

(73) $$\zeta_{v}=\frac{v_{\text{B̶}}}{c}=\frac{100}{\sqrt{1+\frac{3.27817\times 10^{6}}{{\left(E_{\text{bond}}[\text{kcal}/\text{mol}]\right)}^{2}{\left(X_{\text{bond}}[\overset{0}{A}]\right)}^{2}}}}[\%]$$

Next, dealing with a new matter particle, one will be interested also on its charge, respecting the benchmarking charge of an electron. To this end, one re-employs the step (xv) of bondonic algorithm, Equation (11), in the form emphasizing the bondonic charge appearance, namely

(74) $${ℵ}_{\text{B̶}}(e_{\text{B̶}})={ℵ}_{0}$$

Next, when considering for the left-hand side of (74), the form provided by Equation (35), and for the right-hand side of (74), the fundamental hyperfine value of Equation (6), one gets the working Equation

(75) $$c\frac{m_{\text{B̶}}v_{\text{B̶}}}{e_{\text{B̶}}}X_{\text{bond}}=137.036\left[\frac{\text{Joule}\;\times \;\text{meter}}{\text{Coulomb}}\right]$$

from where the bondonic charge appears immediately, once the associate expressions for mass and velocity are considered from Equations (67) and (72), respectively, yielding the quantified form

(76) $$e_{\text{B̶}}=\frac{4\pi \hbar c}{137.036}-\frac{1}{\sqrt{1+\frac{64\pi^{2}E_{\text{bond}}^{2}X_{\text{bond}}^{2}}{\hbar^{2}c^{2}{\left(2\pi n+1\right)}^{4}}}},\;n=0,1,2\ldots$$

However, even for the ground state, and more so for the excited states, one may see that when forming the practical ratio respecting the unitary electric charge from (76), it actually approaches a referential value, namely

(77) $$\zeta_{e}=\frac{e_{\text{B̶}}}{e}=\frac{4\pi }{\sqrt{1+\frac{{\left(E_{\text{bond}}[\text{kcal}/\text{mol}]\right)}^{2}{\left(X_{\text{bond}}[\overset{0}{A}]\right)}^{2}}{3.27817\times 10^{6}{\left(2\pi n+1\right)}^{4}}}}≅4\pi$$

for, in principle, any common energy and length of chemical bonding. On the other side, for the bondons to have different masses and velocities (kinetic energy) as associated with specific bonding energy but an invariant (universal) charge seems a bit paradoxical. Moreover, it appears that with Equation (77) the predicted charge of a bonding, even in small molecules such as H_2_, considerably surpasses the available charge in the system, although this may be eventually explained by the continuous matter-antimatter balance in the Dirac Sea to which the present approach belongs. However, to circumvent such problems, one may further use the result (77) and map it into the Poisson type charge field Equation

(78) $$e_{\text{B̶}}≅4\pi \times e↔\nabla^{2}V≅4\pi \times \rho$$

from where the bondonic charge may be reshaped by appropriate dimensional scaling in terms of the bounding parameters (*E_bond_* and *X_bond_*) successively as

(79) $$e_{\text{B̶}}∼\frac{1}{4\pi }{\left[\nabla_{X}^{2}V\right]}_{X=X_{\text{bond}}}\to \frac{1}{4}\frac{E_{\text{bond}}X_{\text{bond}}}{{ℵ}_{0}}$$

Now, Equation (79) may be employed towards the working ratio between the bondonic and electronic charges in the ground state of bonding

(80) $$\zeta_{e}=\frac{e_{\text{B̶}}}{e}∼\frac{1}{32\pi }\frac{(E_{\text{bond}}[k\;cal/\text{mol}])\left(X_{\text{bond}}[\overset{0}{A}]\right)}{\sqrt{3.27817}\times 10^{3}}$$

With Equation (80) the situation is reversed compared with the previous paradoxical situation, in the sense that now, for most chemical bonds (of Table 1, for instance), the resulted bondonic charge is small enough to be not yet observed or considered as belonging to the bonding wave spreading among the binding electrons.

Instead, aiming to explore the specific information of bonding reflected by the bondonic mass and velocity, the associated ratios of Equations (68) and (73) for some typical chemical bonds [71,72] are computed in Table 1. They may be eventually accompanied by the predicted life-time of corresponding bondons, obtained from the bondonic mass and velocity working expressions (68) and (73), respectively, throughout the basic time-energy Heisenberg relationship—here restrained at the level of kinetic energy only for the bondonic particle; this way one yields the successive analytical forms

(81) $$t_{\text{B̶}}=\frac{\hbar }{T_{\text{B̶}}}=\frac{2\hbar }{m_{\text{B̶}}v_{\text{B̶}}^{2}}=\frac{2\hbar }{(m_{0}\zeta_{m}){\left(c\zeta_{v}\cdot 10^{-2}\right)}^{2}}=\frac{\hbar }{m_{0}c^{2}}\frac{2\cdot 10^{4}}{\zeta_{m}\zeta_{v}^{2}}=\frac{0.0257618}{\zeta_{m}\zeta_{v}^{2}}\times 10^{-15}{\left[s\right]}_{SI}$$

and the specific values for various bonding types that are displayed in Table 1. Note that defining the bondonic life-time by Equation (81) is the most adequate, since it involves the basic bondonic (particle!) information, mass and velocity; instead, when directly evaluating the bondonic life-time by only the bonding energy one deals with the working formula

(82) $$t_{\text{bond}}=\frac{\hbar }{E_{\text{bond}}}=\frac{1.51787}{E_{\text{bond}}[\text{kcal}/\text{mol}]}\times 10^{-14}{\left[s\right]}_{SI}$$

that usually produces at least one order lower values than those reported in Table 1 upon employing the more complex Equation (81). This is nevertheless reasonable, because in the last case no particle information was considered, so that the Equation (82) gives the time of the associate *wave* representation of bonding; this departs by the case when the time is computed by Equation (81) where the information of bonding is contained within the *particle* (bondonic) mass and velocity, thus predicting longer life-times, and consequently a more susceptible timescale in allowing the bondonic observation. Therefore, as far as the chemical bonding is modeled by associate bondonic particle, the specific time of Equation (81) rather than that of Equation (82) should be considered.

While analyzing the values in Table 1, it is generally observed that as the bondonic mass is large as its velocity and the electric charge lower in their ratios, respecting the light velocity and electronic benchmark charge, respectively, however with some irregularities that allows further discrimination in the sub-bonding types. Yet, the life-time tendency records further irregularities, due to its complex and reversed bondonic mass-velocity dependency of Equation (81), and will be given a special role in bondonic observation—see the Table 2 discussion below. Nevertheless, in all cases, the bondonic velocity is a considerable (non-negligible) percent of the photonic velocity, confirming therefore its combined quantum-relativistic nature. This explains why the bondonic reality appears even in the *non-relativistic* case of the Schrödinger equation when augmented with Bohmian entangled motion through the hidden quantum interaction.

Going now to particular cases of chemical bonding in Table 1, the hydrogen molecule maintains its special behavior through providing the bondonic mass as slightly more than double of the only two electrons contained in the whole system. This is not a paradox, but a confirmation of the fact the bondonic reality is not just the sum or partition of the available valence atomic electrons in molecular bonds, but a distinct (although related) existence that fully involves the undulatory nature of the electronic and nuclear motions in producing the chemical field. Remember the chemical field was associated either in Schrödinger as well in Dirac pictures with the internal rotations of the (Bohmian) wave function or spinors, being thus merely a phase property—thus inherently of undulatory nature. It is therefore natural that the risen bondons in bonding preserve the wave nature of the chemical field traveling the bond length distance with a significant percent of light.

Moreover, the bondonic mass value may determine the kind of chemical bond created, in this line the H_2_ being the most covalent binding considered in Table 1 since it is most closely situated to the electronic pairing at the mass level. The excess in H_2_ bond mass with respect to the two electrons in isolated H atoms comes from the nuclear motion energy converted (relativistic) and added to the two-sided electronic masses, while the heavier resulted mass of the bondon is responsible for the stabilization of the formed molecule respecting the separated atoms. The H_2_ bondon seems to be also among the less circulated ones (along the bondon of the F_2_ molecule) in bonding traveled information due to the low velocity and charge record—offering therefore another criterion of covalency, *i.e.*, associated with better localization of the bonding space.

The same happens with the C–C bonding, which is predicted to be more *covalent* for its simple (single) bondon that moves with the *smallest velocity* (ς*_v_*<<) or fraction of the light velocity from all C–C types of bonding; in this case also the bondonic *highest mass* (ς*_m_*>>), *smallest charge* (ς*_e_*<<), and *highest (observed) life-time* (*t_B̶_*>>) criteria seem to work well. Other bonds with high covalent character, according with the bondonic velocity criterion only, are present in N≡N and the C=O bonding types and less in the O=O and C–O ones. Instead, one may establish the criteria for *multiple* (double and triple) *bonds* as having the series of current bondonic properties as: {ς*_m_* <, ς*_v_* >, ς*_e_* >, *t_B̶_* <}

However, the diamond C–C bondon, although with the smallest recorded mass (ς*_m_* <<), is characterized by the highest velocity (ς*_v_* >) and charge (ς*_e_* >) in the CC series (and also among all cases of Table 1). This is an indication that the bond is very much delocalized, thus recognizing the solid state or *metallic* crystallized structure for this kind of bond in which the electronic pairings (the bondons) are distributed over all atomic centers in the unit cell. It is, therefore, a special case of bonding that widely informs us on the existence of conduction bands in a solid; therefore the metallic character generally associated with the bondonic series of properties {ς*_m_* <<, ς*_v_* >, ς*_e_* >, *t_B̶_*<}, thus having similar trends with the corresponding properties of multiple bonds, with the only particularity in the lower mass behavior displayed—due to the higher delocalization behavior for the associate bondons.

Very interestingly, the series of C–H, N–H, and O–H bonds behave similarly among them since displaying a shrink and medium range of mass (moderate high), velocity, charge and life-time (moderate high) variations for their bondons, {ς*_m_* ∼ >, ς*_v_* ∼, ς*_e_* ∼, *t_B̶_* ∼>}; this may explain why these bonds are the most preferred ones in DNA and genomic construction of proteins, being however situated towards the *ionic character* of chemical bond by the lower bondonic velocities computed; they have also the most close bondonic mass to unity; this feature being due to the manifested polarizability and inter-molecular effects that allows the 3D proteomic and specific interactions taking place.

Instead, along the series of halogen molecules F_2_, Cl_2_, and I_2_, only the observed life-time of bondons show high and somehow similar values, while from the point of view of velocity and charge realms only the last two bonding types display compatible properties, both with drastic difference for their bondonic mass respecting the F–F bond—probably due the most negative character of the fluorine atoms. Nevertheless, judging upon the higher life-time with respect to the other types of bonding, the classification may be decided in the favor of covalent behavior. At this point, one notes traces of covalent bonding nature also in the case of the rest of halogen-carbon binding (C–Cl, C–Br, and C–I in Table 1) from the bondonic life-time perspective, while displaying also the ionic manifestation through the velocity and charge criteria {ς*_v_* ∼, ς*_e_* ∼} and even a bit of metal character by the aid of small bondonic mass (ς*_m_* <). All these mixed features may be because of the joint existence of both inner electronic shells that participate by electronic induction in bonding as well as electronegativity difference potential.

Remarkably, the present results are in accordance with the recent signalized new binding class between the electronic pairs, somehow different from the ionic and covalent traditional ones in the sense that it is seen as a kind of resonance, as it appears in the molecular systems like F_2_, O_2_, N_2_ (with impact in environmental chemistry) or in polar compounds like C–F (specific to ecotoxicology) or in the reactions that imply a competition between the exchange in the hydrogen or halogen (e.g., HF). The valence explanation relied on the possibility of higher orders of orbitals’ existing when additional shells of atomic orbitals are involved such as <f> orbitals reaching this way the *charge-shift bonding* concept [73]; the present bondonic treatment of chemical bonds overcomes the charge shift paradoxes by the relativistic nature of the bondon particles of bonding that have as inherent nature the time-space or the energy-space spanning towards electronic pairing stabilization between centers of bonding or atomic adducts in molecules.

However, we can also made predictions regarding the values of bonding energy and length required for a bondon to acquire either the unity of electronic charge or its mass (with the consequence in its velocity fraction from the light velocity) on the ground state, by setting Equations (68) and (80) to unity, respectively. These predictions are summarized in Table 2.

From Table 2, one note is that the situation of the bondon having the same charge as the electron is quite improbable, at least for the common chemical bonds, since in such a case it will feature almost the light velocity (and almost no mass–that is, however, continuously decreasing as the bonding energy decreases and the bonding length increases). This is natural since a longer distance has to be spanned by lower binding energy yet carrying the same unit charge of electron while it is transmitted with the same relativistic velocity! Such behavior may be regarded as the present *zitterbewegung* (trembling in motion) phenomena, here at the bondonic level. However one records the systematic increasing of bondonic life-time towards being observable in the femtosecond regime for increasing bond length and decreasing the bonding energy–under the condition the chemical bonding itself still exists for certain {*X_bond_*, *E_bond_*} combinations.

On the other side, the situation in which the bondon will weigh as much as one electron is a current one (see the Table 1); nevertheless, it is accompanied by quite reasonable chemical bonding length and energy information that it can carried at a low fraction of the light velocity, however with very low charge as well. Nevertheless, the discovered bonding energy-length relationship from Table 2, based on Equation (80), namely

(83) $$E_{\text{bond}}[\text{kcal}/\text{mol}]\times X_{\text{bond}}[\overset{0}{A}]=182019$$

should be used in setting appropriate experimental conditions in which the bondon particle *B̶* may be observed as carrying the unit electronic charge yet with almost zero mass. In this way, *the bondon is affirmed as a special particle of Nature, that when behaving like an electron in charge it is behaving like a photon in velocity and like neutrino in mass, while having an observable (at least as femtosecond) lifetime for nanosystems having chemical bonding in the range of hundred of Angstroms and thousands of kcal/mol*! Such a peculiar nature of a bondon as the quantum particle of chemical bonding, the central theme of Chemistry, is not as surprising when noting that Chemistry seems to need both a particle view (such as offered by relativity) and a wave view (such as quantum mechanics offers), although nowadays these two physics theories are not yet fully compatible with each other, or even each fully coherent internally. Maybe the concept of ‘bondons’ will help to improve the situation for all concerned by its further conceptual applications.

Finally, just to give a conceptual glimpse of how the present bondonic approach may be employed, the scattering phenomena are considered within its Raman realization, viewed as a sort of generalized Compton scattering process, *i.e.*, extracting the structural information from various systems (atoms, molecules, crystals, *etc*.) by modeling the inelastic interaction between an incident IR photon and a quantum system (here the bondons of chemical bonds in molecules), leaving a scattered wave with different frequency and the resulting system in its final state [74]. Quantitatively, one firstly considers the interaction Hamiltonian as being composed by two parts,

(84) $$H^{\left(1\right)}=-\frac{e_{\text{B̶}}}{m_{\text{B̶}}}\sum_{j}\left[{\vec{p}}_{\text{B̶}j}\cdot \vec{A}({\vec{r}}_{j},t)\right]$$

(85) $$H^{\left(2\right)}=\frac{e_{\text{B̶}}^{2}}{2m_{\text{B̶}}}\sum_{j}{\vec{A}}^{2}({\vec{r}}_{j},t)$$

accounting for the linear and quadratic dependence of the light field potential vector *A⃗*(*r⃗_j_*, *t*) acting on the bondons “*j*”, carrying the kinetic moment *p_B̶j_* = *m_B̶_v_B̶_*, charge *e_B̶_* and mass *m_B̶._*

**Figure 1.**
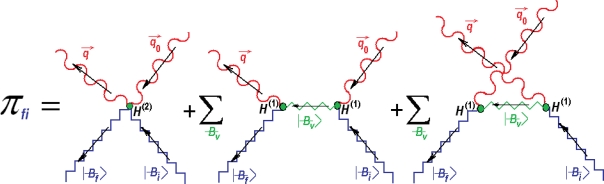
The Feynman diagrammatical sum of interactions entering the Raman effect by connecting the single and double photonic particles’ events in absorption (incident wave light *q⃗*_0_, υ_0_) and emission (scattered wave light *q⃗*, υ) induced by the quantum first *H*^(1)^ and second *H*^(2)^ order interaction Hamiltonians of Equations (84) and (85) through the initial |*B̶_i_* 〉, final |*B̶_f_* 〉, and virtual |*B̶_v_* 〉 bondonic states. The first term accounts for absorption (*A*)-emission (*E*) at once, the second term sums over the virtual states connecting the absorption followed by emission, while the third terms sums over virtual states connecting the absorption following the emission events.

Then, noting that, while considering the quantified incident (*q⃗*_0_, υ_0_) and scattered (*q⃗*, υ) light beams, the interactions driven by *H*^(1)^ and *H*^(2)^ model the changing in one- and two- occupation numbers of photonic trains, respectively. In this context, the transition probability between the initial |*B̶_i_* 〉 and final |*B̶_f_* 〉 bondonic states writes by squaring the sum of all scattering quantum probabilities that include absorption (*A*, with *n_A_* number of photons) and emission (*E*, with *n_E_* number of photons) of scattered light on bondons, see Figure 1.

Analytically, one has the *initial-to-final* total transition probability [75]dependence here given as

(86) $$\begin{array}{c} d^{2}\Pi_{fi}∼\frac{1}{\hbar }{\left|\pi_{fi}\right|}^{2}\delta \left(E_{|{\text{B̶}}_{i}\rangle }+h\upsilon_{0}-E_{|{\text{B̶}}_{f}\rangle }-h\upsilon \right)\upsilon^{2}\;\;d\upsilon d\Omega \\ =\frac{1}{\hbar }|\langle f;n_{A}-1,\;n_{E}+1\left|H^{\left(2\right)}\right|n_{A},\;n_{E};i\rangle \\ +\sum_{{\text{B̶}}_{v}}\frac{\langle {\text{B̶}}_{f};n_{A}-1,\;n_{E}+1\left|H^{\left(1\right)}\right|n_{A}-1,\;n_{E};{\text{B̶}}_{v}\rangle \langle {\text{B̶}}_{v};n_{A}-1,\;n_{E}\left|H^{\left(1\right)}\right|n_{A},\;n_{E};{\text{B̶}}_{i}\rangle }{E_{|{\text{B̶}}_{i}\rangle }-E_{|{\text{B̶}}_{v}\rangle }+h\upsilon_{0}}\\ +\sum_{{\text{B̶}}_{v}}\frac{\langle {\text{B̶}}_{f};n_{A}-1,\;n_{E}+1\left|H^{\left(1\right)}\right|n_{A},\;n_{E}+1;{\text{B̶}}_{v}\rangle \langle {\text{B̶}}_{v};n_{A},\;n_{E}+1\left|H^{\left(1\right)}\right|n_{A},\;n_{E};{\text{B̶}}_{i}\rangle }{E_{|{\text{B̶}}_{i}\rangle }-E_{|{\text{B̶}}_{v}\rangle }-h\upsilon }{|}^{2}\\ \times \delta \left(E_{|{\text{B̶}}_{i}\rangle }+h\upsilon_{0}-E_{|{\text{B̶}}_{f}\rangle }-h\upsilon \right)\upsilon^{2}\;d\upsilon d\Omega \end{array}$$

At this point, the conceptual challenge appears to explore the existence of the Raman process itself from the bondonic description of the chemical bond that turns the incoming IR photon into the (induced, stimulated, or spontaneous) structural frequencies

(87) $$\upsilon_{v\leftarrow i}=\frac{E_{|{\text{B̶}}_{i}\rangle }-E_{|{\text{B̶}}_{v}\rangle }}{h}$$

As such, the problem may be reshaped in expressing the virtual state energy *E*_*|B̶*_*v*_ 〉_ in terms of bonding energy associated with the initial state

(88) $$E_{|{\text{B̶}}_{i}\rangle }=E_{\text{bond}}$$

that can be eventually measured or computationally predicted by other means. However, this further implies the necessity of expressing the incident IR photon with the aid of bondonic quantification; to this end the Einstein relation (69) is appropriately reloaded in the form

(89) $$h\upsilon_{v\leftarrow i}=\frac{m_{\text{B̶}}v_{\text{B̶}}^{2}}{2}=\frac{1}{4}\frac{v_{\text{B̶}}^{2}\hbar^{2}}{E_{\text{bond}}X_{\text{bond}}^{2}}{\left(2\pi n_{v}+1\right)}^{2}$$

where the bondonic mass (67) was firstly implemented. Next, in terms of representing the turn of the incoming IR photon into the structural *wave*-frequency related with the bonding energy of initial state, see Equation (88); the time of wave-bond (82) is here considered to further transform Equation (89) to the yield

(90) $$h\upsilon_{v\leftarrow i}=\frac{1}{4}\frac{v_{\text{B̶}}^{2}E_{\text{bond}}^{2}t_{\text{bond}}^{2}}{E_{\text{bond}}X_{\text{bond}}^{2}}{\left(2\pi n_{v}+1\right)}^{2}=\frac{1}{4}E_{\text{bond}}\frac{v_{\text{B̶}}^{2}}{v_{\text{bond}}^{2}}{\left(2\pi n_{v}+1\right)}^{2}$$

where also the corresponding wave-bond velocity was introduced

(91) $$v_{\text{bond}}=\frac{X_{\text{bond}}}{t_{\text{bond}}}=\frac{1}{\hbar }E_{\text{bond}}X_{\text{bond}}$$

It is worth noting that, as previously was the case with the dichotomy between bonding and bondonic times, sees Equations (81)*vs*. (82), respectively, the bonding velocity of Equation (91) clearly differs by the bondonic velocity of Equation (72) since the actual working expression

(92) $$\frac{v_{\text{bond}}}{c}=(E_{\text{bond}}[\text{kcal}/\text{mol}])\left(X_{\text{bond}}[\overset{0}{A}]\right)2.19758\times 10^{-3}[\%]$$

provides considerably lower values than those listed in Table 1–again, due to missing the inclusion of the particle mass’ information, unlike is the case for the bondonic velocity.

Returning to the bondonic description of the Raman scattering, one replaces the virtual photonic frequency of Equation (90) together with Equation (88) back in the Bohr-type Equation (87) to yield the searched quantified form of virtual bondonic energies in Equation (86) and Figure 1, analytically

(93) $$\begin{array}{c} E_{|{\text{B̶}}_{v}\rangle }=E_{\text{bond}}\left[1-\frac{1}{4}\frac{v_{\text{B̶}}^{2}}{v_{\text{bond}}^{2}}{\left(2\pi n_{v}+1\right)}^{2}\right]\\ =E_{\text{bond}}\left[1-16\pi^{2}\frac{{\left(2\pi n_{v}+1\right)}^{2}}{64\pi^{2}\frac{E_{\text{bond}}^{2}X_{\text{bond}}^{2}}{\hbar^{2}c^{2}}+{\left(2\pi n_{v}+1\right)}^{4}}\right] \end{array}$$

or numerically

(94) $$E_{|{\text{B̶}}_{v}\rangle }=E_{\text{bond}}\left[1-\frac{16\pi^{2}{\left(2\pi n_{v}+1\right)}^{2}}{0.305048\times 10^{-6}\times {\left(E_{\text{bond}}[\text{kcal}/\text{mol}]\right)}^{2}\times {\left(X_{\text{bond}}[\overset{0}{A}]\right)}^{2}+{\left(2\pi n_{v}+1\right)}^{4}}\right],\;n_{v}=0,1,2\ldots$$

Remarkably, the bondonic quantification (94) of the virtual states of Raman scattering varies from negative to positive energies as one moves from the ground state to more and more excited states of initial bonding state approached by the incident IR towards virtual ones, as may be easily verified by considering particular bonding data of Table 1. In this way, more space is given for future considerations upon the inverse or stimulated Raman processes, proving therefore the direct involvement of the bondonic reality in combined scattering of light on chemical structures.

Overall, the bondonic characterization of the chemical bond is fully justified by quantum and relativistic considerations, to be advanced as a useful tool in characterizing chemical reactivity, times of reactions, *i.e.*, when tunneling or entangled effects may be rationalized in an analytical manner.

Note that further correction of this bondonic model may be realized when the present point-like approximation of nuclear systems is abolished and replaced by the bare-nuclear assumption in which additional dependence on the bonding distance is involved. This is left for future communications.

## 5. Conclusion

The chemical bond, perhaps the greatest challenge in theoretical chemistry, has generated many inspiring theses over the years, although none definitive. Few of the most preeminent regard the orbitalic based explanation of electronic pairing, in valence shells of atoms and molecules, rooted in the hybridization concept [8] then extended to the valence-shell electron-pair repulsion (VSEPR) [76]. Alternatively, when electronic density is considered, the atoms-in-molecule paradigms were formulated through the geometrical partition of forces by Berlin [69], or in terms of core, bonding, and lone-pair lodges by Daudel [77], or by the zero local flux in the gradient field of the density ∇ρ by Bader [26], until the most recent employment of the chemical action functional in bonding [78,79].

Yet, all these approaches do not depart significantly from the undulatory nature of electronic motion in bonding, either by direct wave-function consideration or through its probability information in electronic density manifestation (for that is still considered as a condensed—observable version—of the undulatory manifestation of electron).

In other words, while passing from the Lewis point-like ansatz to the undulatory modeling of electrons in bonding, the reverse passage was still missing in an analytical formulation. Only recently the first attempt was formulated, based on the broken-symmetry approach of the Schrödinger Lagrangean with the electronegativity-chemical hardness parabolic energy dependency, showing that a systematical quest for the creation of particles from the chemical bonding fields is possible [80].

Following this line, the present work makes a step forward and considers the gauge transformation of the electronic wave-function and spinor over the de Broglie-Bohm augmented non-relativistic and relativistic quantum pictures of the Schrödinger and Dirac electronic (chemical) fields, respectively. As a consequence, the reality of the chemical field in bonding was proved in either framework, while providing the corresponding bondonic particle with the associate mass and velocity in a full quantization form, see Equations (67) and (72). In fact, the Dirac bondon (65) was found to be a natural generalization of the Schrödinger one (38), while supplementing it with its anti-bondon particle (66) for the positron existence in the Dirac Sea.

The bondon is the quantum particle corresponding to the superimposed electronic pairing effects or distribution in chemical bond; accordingly, through the values of its mass and velocity it may be possible to indicate the type of bonding (in particular) and the characterization of electronic behavior in bonding (in general).

However, one of the most important consequences of bondonic existence is that the chemical bonding may be described in a more complex manner than relaying only on the electrons, but eventually employing the fermionic (electronic)-bosonic (bondonic) mixture: the first preeminent application is currently on progress, that is, exploring the effect that the Bose-Einstein condensation has on chemical bonding modeling [81,82]. Yet, such possibility arises due to the fact that whether the Pauli principle is an independent axiom of quantum mechanics or whether it depends on other quantum description of matter is still under question [83], as is the actual case of involving hidden variables and the entanglement or non-localization phenomenology that may be eventually mapped onto the delocalization and fractional charge provided by quantum chemistry over and on atomic centers of a molecular complex/chemical bond, respectively.

As an illustration of the bondonic concept and of its properties such as the mass, velocity, charge, and life-time, the fundamental Raman scattering process was described by analytically deriving the involved virtual energy states of scattering sample (chemical bond) in terms of the bondonic properties above—proving its necessary existence and, consequently, of the associate Raman effect itself, while leaving space for further applied analysis based on spectroscopic data on hand.

On the other side, the mass, velocity, charge, and life-time properties of the bondons were employed for analyzing some typical chemical bonds (see Table 1), this way revealing a sort of fuzzy classification of chemical bonding types in terms of the bondonic-to-electronic mass and charge ratios ς*_m_* and ς*_e_*, and of the bondonic-to-light velocity percent ratio ς*_v_*, along the bondonic observable life-time, *t_B̶_* respectively–here summarized in Table 3.

These rules are expected to be further refined through considering the new paradigms of special relativity in computing the bondons’ velocities, especially within the modern algebraic chemistry [84]. Yet, since the bondonic masses of chemical bonding ground states seem untouched by the Dirac relativistic considerations over the Schrödinger picture, it is expected that their analytical values may make a difference among the various types of compounds, while their experimental detection is hoped to be some day completed.

## Figures and Tables

**Table 1.** Ratios for the bondon-to-electronic mass and charge and for the bondon-to-light velocity, along the associated bondonic life-time for typical chemical bonds in terms of their basic characteristics such as the bond length and energy [71,72] through employing the basic formulas (68), (73), (80) and (81) for the ground states, respectively.

**Bond Type**	*X_bond_***(Å)**	*E_bond_***(kcal/mol)**	$\zeta_{m}=\frac{m_{\text{B̶}}}{m_{0}}$	$\zeta_{v}=\frac{v_{\text{B̶}}}{c}[\%]$	$\zeta_{e}=\frac{e_{\text{B̶}}}{e}[\times 10^{3}]$	*t_B̶_*[×10^15^] **(seconds)**
**H–H**	0.60	104.2	2.34219	3.451	0.3435	9.236

**C–C**	1.54	81.2	0.45624	6.890	0.687	11.894
**C–C (in diamond)**	1.54	170.9	0.21678	14.385	1.446	5.743

**C=C**	1.34	147	0.33286	10.816	1.082	6.616
**C≡C**	1.20	194	0.31451	12.753	1.279	5.037

**N≡N**	1.10	225	0.32272	13.544	1.36	4.352
**O=O**	1.10	118.4	0.61327	7.175	0.716	8.160

**F–F**	1.28	37.6	1.42621	2.657	0.264	25.582
**Cl–Cl**	1.98	58	0.3864	6.330	0.631	16.639
**I–I**	2.66	36.1	0.3440	5.296	0.528	26.701

**C–H**	1.09	99.2	0.7455	5.961	0.594	9.724
**N–H**	1.02	93.4	0.9042	5.254	0.523	10.32
**O–H**	0.96	110.6	0.8620	5.854	0.583	8.721

**C–O**	1.42	82	0.5314	6.418	0.64	11.771

**C=O (in CH**_**2**_**O)**	1.21	166	0.3615	11.026	1.104	5.862
**C=O (in O=C=O)**	1.15	191.6	0.3467	12.081	1.211	5.091

**C–Cl**	1.76	78	0.3636	7.560	0.754	12.394
**C–Br**	1.91	68	0.3542	7.155	0.714	14.208
**C–I**	2.10	51	0.3906	5.905	0.588	18.9131

**Table 2.** Predicted basic values for bonding energy and length, along the associated bondonic life-time and velocity fraction from the light velocity for a system featuring unity ratios of bondonic mass and charge, respecting the electron values, through employing the basic formulas (81), (73), (68), and (80), respectively.

$X_{\text{bond}}[\overset{0}{A}]$	*E_bond_* [(*kcal/mol*)]	*t_B̶_*[×10^15^] (seconds)	$\zeta_{v}=\frac{v_{\text{B̶}}}{c}[\%]$	$\zeta_{m}=\frac{m_{\text{B̶}}}{m_{0}}$	$\zeta_{e}=\frac{e_{\text{B̶}}}{e}$
**1**	**87.86**	10.966	4.84691	1	0.4827 × 10^−3^
**1**	**182019**	53.376	99.9951	4.82699 × 10^−4^	1
**10**	**18201.9**	533.76	99.9951	4.82699 × 10^−5^	1
**100**	**1820.19**	5337.56	99.9951	4.82699 × 10^−6^	1

**Table 3.** Phenomenological classification of the chemical bonding types by bondonic (mass, velocity, charge and life-time) properties abstracted from Table 1; the used symbols are: > and ≫ for ‘high’ and ‘very high’ values; < and ≪ for ‘low’ and ‘very low’ values; ∼ and ∼> for ‘moderate’ and ‘moderate high and almost equal’ values in their class of bonding.

**Property**		ς*_m_*	ς*_v_*	ς*_e_*	*t_B̶_*
**Chemical bond**					
Covalence		>>	<<	<<	>>
Multiple bonds		<	>	>	<
Metallic		<<	>	>	<
Ionic		∼>	∼	∼	∼>
